# HbS Binding to GP1bα Activates Platelets in Sickle Cell Disease

**DOI:** 10.1371/journal.pone.0167899

**Published:** 2016-12-09

**Authors:** Gowtham K. Annarapu, Rashi Singhal, Avinash Gupta, Sheetal Chawla, Harish Batra, Tulika Seth, Prasenjit Guchhait

**Affiliations:** 1 Disease Biology Laboratory, Regional Centre for Biotechnology, National Capital Region Biotech Science Cluster, Faridabad, India; 2 Department of Biotechnology, Manipal University, Karnataka, India; 3 Department of Medicine-Hematology, All India Institute of Medical Sciences, New Delhi, India; Royal College of Surgeons in Ireland, IRELAND

## Abstract

Intravascular hemolysis increases the risk of thrombosis in hemolytic disorders. Our previous study showed that the binding of adult hemoglobin (HbA) to glycoprotein (GP) 1bα induced the activation of platelets. The elevated plasma Hb or platelet surface bound Hb positively correlated with platelet activation in patients with paroxysmal nocturnal hemoglobinuria (PNH). Furthermore, this study shows that the sickle Hb [HbS, occurs due to single nucleotide polymorphism at A>T of β-globin gene of Hb and causes sickle cell disease (SCD)] also bound to GP1bα and activated platelets in a concentration-dependent manner. The HbS bound to glycocalicin (extramembranous part of GP1bα) with K_D_ ~ 10.46 ± 3 μM. HbS induced phosphorylation of signaling adapter proteins, such as Lyn, PI3K, Akt and ERK in platelets, and also increased the surface expression of platelet activation markers such as P-selectin (10.7 fold) and PAC1 binding (10.4 fold) in platelet surface in a concentration-dependent manner. HbS also increased the platelet microparticle-generation (4.7 fold) and thrombus-formation (4.3 fold) in a concentration-dependent manner. An elevated level of extracellular Hb in plasma correlated directly with platelet activation markers such as P-selectin (r = 0.7947), PAC1 binding (r = 0.5914) on platelet surface and plasma levels of platelet-derived microparticles (r = 0.7834) in patients with SCD. Our study therefore suggests that the HbS-induced platelet activation may play a crucial role in intravascular clot formation observed in SCD patients characterized by high propensity to vascular occlusion and hypercoagulable states.

## Introduction

Sickle cell disease (SCD) is a hemolytic disorder caused by the single nucleotide substitution homozygous mutation (A>T) at β-globin gene of hemoglobin (HbS, changing the amino acid Glu>Val at the 6^th^ position) [1, 2] that affects millions of people around the world. An estimated 2% of the world’s population carries HbS gene, and about 3 million infants are born every year with SCD [3, 4]. SCD is characterized by sickle shaped red blood cells, chronic intravascular hemolysis and high propensity to vasoocclusive crisis [2, 5]. The vascular occlusion and related clinical events such as ischemic attacks and strokes cause significant morbidity estimating one third of total deaths in SCD [6]. Besides, other clinical complications such as hypercoagulable states and thrombosis are also considered as leading causes of death in these patients [7, 8].

Several studies have revealed that platelet activation; hypercoagulation and thrombosis contribute significantly to vessel occlusion in SCD [2, 9]. The major factor that contributes to the platelet activation in SCD is abundance of free Hb in circulation, which alters platelet functions by limiting the bioavailability of nitric oxide (NO) [8, 10, 11]. It has been reported that NO inhibits platelet aggregation and adhesion to subendothelium matrices/endothelium via cyclic guanosine monophosphate (GMP) pathway [8, 9, 12]. Studies also have described the stimulatory role of intraerythrocytic ADP in platelet activation during intravascular hemolysis [11]. Besides, our recent study shows that Hb, specifically adult Hb (HbA) can bind directly to GP1bα on platelet surface and induce its activation. An elevated level of free Hb in plasma correlates directly with platelet activation in patients with PNH [13]. This study further reveals that HbS also binds directly to GP1bα on platelets and modulates its activation. The HbS-mediated activation of platelets promotes thrombus formation *in vitro*. The elevated plasma Hb directly correlates with platelet activation in patients with SCD.

## Materials and Methods

### Materials

Antibodies, phospho (p)-Lyn, p-PI3K, p-Akt (S473), p-ERK, and Lyn, PI3K, Akt and ERK were purchased from Cell Signaling (Beverly, MA, USA). Goat anti-rabbit/mouse IgG conjugated with peroxidase were purchased from Pierce (Rockford, IL, USA). The fluorescence antibodies, anti-human P-selectin FITC (R&D systems, Minneapolis), anti-human CD41a PE, PAC1 FITC and Annexin V FITC (BD Pharmingen™ (BD Biosciences), were purchased. Anti-hemoglobin α was purchased from Santa Cruz Biotechnology (Santa Cruz, CA, USA). The synthetic peptides (designed from N-terminal region of GP1bα) including AA1-50 (mpllllllllpsplhphpicwvskvashlevncdkrnltalppdlpkdtt) and scrambled control peptide (sctvrltknhdadedtlalppklnmvlplseilchlplvpsklplhllpp) were purchased from (GL BioChem, Shanghai). These peptides have been used successfully in our recent studies [13, 14]. Majority of other laboratory chemicals including hemoglobin S (HbS with a purity of 98.5% isolated through Sephadex G-25 column (gel filtration chromatography) followed by ion exchange using CM-sepharose fast flow) were purchased from Sigma-Aldrich, St. Louis, USA. Hemopexin was purchased from Prospec (Prospec, Ness-Ziona, Israel), haptoglobin (Sigma Aldrich, St. Louis, USA) and ADP Colorimetric Assay kit was purchased from BioVision (BioVision,CA, USA).

### Blood samples

To collect blood samples, approval was obtained from the Institutional Ethics Committee (Human Research) of Regional Centre for Biotechnology (RCB, reference No. RCB-IEC-H-2, dated 16^th^ Dec 2013) and All India Institute of Medical Sciences (AIIMS, reference No. IEC-NP-412/2013, dated 4^th^ Sept 2013), Delhi, India. Informed consent was provided according to the recommendations of the declaration of Helsinki. Seventeen patients with SCD and nineteen normal healthy individuals (n = 9 added into calculation, n = 10 were used for isolating platelets and parallel flow assays) were recruited following written consent. 5–10 mL of blood sample was collected in 0.32% sodium citrate anticoagulant or acid-citrate dextrose (ACD) anticoagulant. The patients had been diagnosed for SCD (homozygous) by HPLC fractionation for HbS. The SCD patients were recruited at AIIMS Hematology Department and healthy individuals at RCB.

### Washed platelet preparation

The platelet-rich plasma (PRP) obtained from ACD anticoagulant blood was resuspended in calcium-free tyrode buffer [126 mM NaCl, 2.7 mM KCl, 1 mM MgCl_2_, 0.38 mM NaH_2_PO_4_, 5.6 mM Dextrose, 6.2 mM Sodium HEPES, 8.8 mM HEPES-free acid, 0.1% bovine serum albumin (BSA), pH 6.5]. The PRP was isolated through Sepharose 2B (Sigma-Aldrich, St. Louis, USA) gel filtration column and washed platelets were collected as mentioned in our recent work [13]. The platelets were counted using hemocytometer before assays.

### Glycocalicin purification

Glycocalicin (extramembranous proteolytic fragment of platelet GP1bα) was purified from outdated human platelets according to modified protocols [15, 16]. Briefly, platelets were washed with 13 mM sodium citrate, 120 mM NaCl, 30 mM glucose, pH-7.0, and resuspended in 10 mM tris-HCl, 150 mM NaCl, 2 mM CaCl_2_, pH-7.4 and subjected to sonication. The suspension was incubated at 37°C for 1 hr and processed for ultracentrifugation at 33,000 rpm for 1 hr at 4°C. The supernatant was loaded on to the lectin wheat germ agarose column (Sigma, USA) and eluted with 2.5% N-acetyl-D-glucosamine in 20 mM Tris-HCl, pH-7.4. The eluted fractions were dialyzed with 20 mM Tris-HCl, pH-7.4 and processed for ion exchange chromatography using HiTrap DEAE-FF column (GE Healthcare). Glycocalicin was eluted with a linear salt gradient of 0–0.7 M NaCl in 20 mM Tris-HCl, pH-7.4, and the peak was confirmed by immunoblotting using anti-GP1b SZ2 antibody. The glycocalicin fraction was dialyzed with 25 mM Na_2_HPO_4_, 100 mM NaCl, pH 8.0 and concentrated using Amicon centrifugal tubes (MWCO 30 KDa) following which protein concentration was measured using Pierce BCA protein assay kit.

### Surface plasmon resonance

The Hb binding to glycocalicin was measured using a BIAcore- T200 system (GE Healthcare Life Sciences, USA). The purified glycocalicin was covalently coupled to C1 sensor chip via aldehyde coupling to 1200 resonance units (RU). The binding kinetics assay was performed in 10 mM HEPES, 150 mM NaCl, 0.05% Tween 20, pH 7.4 at 25°C at a flow rate of 30 μL/min. The binding affinity (K_D_) was calculated using steady state affinity binding model, BIAcore T200 evaluation software (v. 2.0) as mentioned [13].

### ELISA

Binding of HbS to glycocalicin was detected using ELISA. ELISA plate was coated with glycocalicin (10μg/mL) and blocked with 2% BSA. Various concentrations of HbS (0.25μM, 0.5 μM, 1 μM, 3 μM and 5 μM) were incubated with immobilized glycocalicin at 37°C for an hour. The bound HbS was detected using goat anti-Hbα antibody (Santa Cruz Biotechnology, Dallas, Texas USA) and HRP tagged donkey anti-goat IgG antibody (Bethyl laboratories, Texas, USA). Further, the binding of HbS to glycocalicin was examined in presence of haptoglobin (Hp) or hemopexin (Hpx) of different concentrations 1 μM, 2.5 μM, and 5 μM.

### Immunoblotting

Washed platelets were incubated with various concentrations of HbS. Platelet pellet was washed and lysed with RIPA buffer in presence of Halt^TM^ protease-phosphatase inhibitor (Thermo Scientific, USA). The lysis products were processed for SDS PAGE and immunoblotted for all above signaling molecules associated with platelet activation including Lyn, PI3K, Akt and ERK (phosphor and non-phospho).

### Flow Cytometry

Flow cytometry was performed to detect the activation markers on platelet surface including P-selectin expression and PAC1 binding. Washed platelets treated with various concentrations of HbS or platelets isolated from patients and controls were labeled with anti-P-selection FITC or PAC1 FITC antibody. The platelet surface expression of above proteins was measured by flow cytometry (Becton Dickinson, San Jose, CA, USA) and expressed as the mean fluorescence.

### Confocal fluorescent microscopy

Anti-P selection FITC antibody was added to washed human platelets (0.5 × 10^6^/mL) and smeared on glass cover slides pre-coated with BSA or HbS, and incubated for 30–45 min at 37°C. The BSA (2%) or HbS (1.5 μM) was incubated on cover slides for 1 hr at 37°C for immobilization. The HbS coated surface was further incubated with 2% BSA for 30 min at RT. After gentle wash with modified Tyrode buffer, slides with adhered-platelets were mounted with Fluoromount-G (Southern Biotech Assoc., Birmingham, AL, USA) and image was captured using a DMI6000B confocal microscope (TCS-SP5, Leica Microsystems, Germany).

### Parallel flow chamber

Platelet thrombus formation assay was performed by perfusing whole blood (collected in citrate-anticoagulant from healthy individuals) over the petri plate immobilized with VWF (plasma purified). The purification of plasma VWF is mentioned in our earlier work [17]. Whole blood was incubated for 15 min with different concentrations of HbS before perfusion. A syringe pump (Harvard Apparatus Inc., USA) was connected to the outlet port that drew blood through the chamber at shear stress of 25 dyne/cm^2^. The flow chamber was mounted onto a Nikon Eclipse Ti-E inverted stage microscope (Nikon, Japan) equipped with a high-speed digital camera. Movie was recorded at magnification 40X and analyzed using NIS-Elements version 4.2 software.

### Measurement of extracellular Hb level in plasma

The Hb concentration in plasma was measured using the sandwich ELISA. Briefly, plasma (100 μL of 1:5,000 dilutions) from patients or normal individuals was incubated in microtiter plates coated with rabbit anti-Hb antibody (Sigma-Aldrich, St. Louis, USA) for 1 hr at RT. The bound Hb was detected by an affinity purified goat anti-Hb conjugated with HRP (Abcam, Cambridge, USA) using TMB substrate at an OD ~ 450 nm. Plasma Hb concentration was calculated as μM compared to human erythrocyte purified HbS (Sigma-Aldrich, St. Louis, USA) as mentioned in our earlier work [13].

### Microparticles measurement from plasma

Plasma microparticles (MPs) were isolated from 5 mL of citrate anti-coagulated plasma. Platelet-free plasma (PFP) was obtained by 3 sequential centrifugation of blood at 700 g for 6 min; platelet-rich plasma at 1500 g for 7 min; and platelet-poor plasma at 1500 g for 15 min. Platelet-derived MPs were measured from PFP in filtered-PBS using anti-CD41 PE antibody and quantified using flow cytometry as mentioned in our previous study [13].

### Statistical analysis

Experimental values were presented as mean ± standard error of mean (SEM) or standard deviation (SD). The Student’s t-test (paired and unpaired), one-way ANOVA and Pearson correlation analysis were used for data analysis and a p-value less than 0.05 was considered to be statistically significant.

## Results

### HbS activates platelets

**Fig 1:**
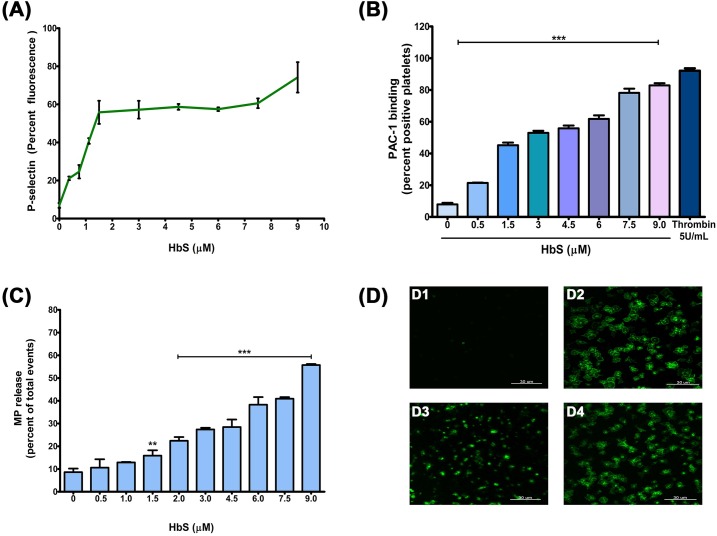
Platelet activation in presence of HbS. **(A)** Washed platelets were incubated with various concentrations of HbS and labeled with anti-P selectin FITC antibody for flow cytometry measurement. Data present the mean ± SEM percent fluorescence from three independent experiments. HbS increased the P-selectin expression in a concentration-dependent manner (P<0.0001). **(B)** The PAC1 binding to platelets GPIIbIIIa was measured using flow cytometry. The HbS increased the PAC1 binding in a concentration-dependent manner, ***P<0.0001. Thrombin used as positive control. **(C)** Microparticle (MP) generation by platelets. Washed platelets were incubated with various concentrations of HbS and the CD41a positive MPs were measured using flow cytometry. The HbS increased the MP generation in a concentration-dependent manner, **P<0.001 and ***P<0.0001 compared to HbS 0 μM. **(D)** Platelet spreading on HbS coated surface. Washed platelets were incubated over surface coated with either BSA (2%) **(D1)** or HbS (1.5 μM) **(D2)**. The platelets were labeled with anti-P selectin FITC antibody and observed under confocal microscope at 63X. The platelet spreading was observed only on HbS surface. The above platelet activation was inhibited by peptide AA1-50 (1 μM) **(D3),** not by control peptide **(D4).**

To investigate the mechanism of platelet activation by sickle Hb, we performed *in vitro* experiments by incubating platelets (freshly isolated from healthy individuals) with different concentrations of erythrocyte-purified HbS. HbS increased the platelet surface P-selectin expression (in percent fluorescence) from 6.90±1.28 (without HbS) to 74.16±7.96 (with 9μM HbS) in a concentration-dependent manner (Fig 1A); and PAC1 binding to GPIIbIIIa (measured in percent positive events) from 7.91±0.96 (without HbS) to 82.89±1.3 (with 9μM HbS) in concentration-dependent manner (Fig 1B). HbS also increased the microparticle generation by platelets (estimated as percent CD41a positive events) from 8.6±1.60 (without HbS) to 40.91±0.69 (with 9μM HbS) in a concentration-dependent manner (Fig 1C). Furthermore, the microscopic image showed the spreading and activation of platelets over HbS coated surface. The inhibition of platelet spreading on immobilized HbS by the peptide AA1-50 (blocks Hb-GP1bα binding) confirmed further the HbS-mediated activation of platelets (Fig 1D). Study also examined the possibility of platelet activation by intraerythrocytic ADP. Since no ADP was detected in HbS stock (S1 Fig) study therefore excludes its effect on platelets.

### HbS binds to glycocalicin

**Fig 2:**
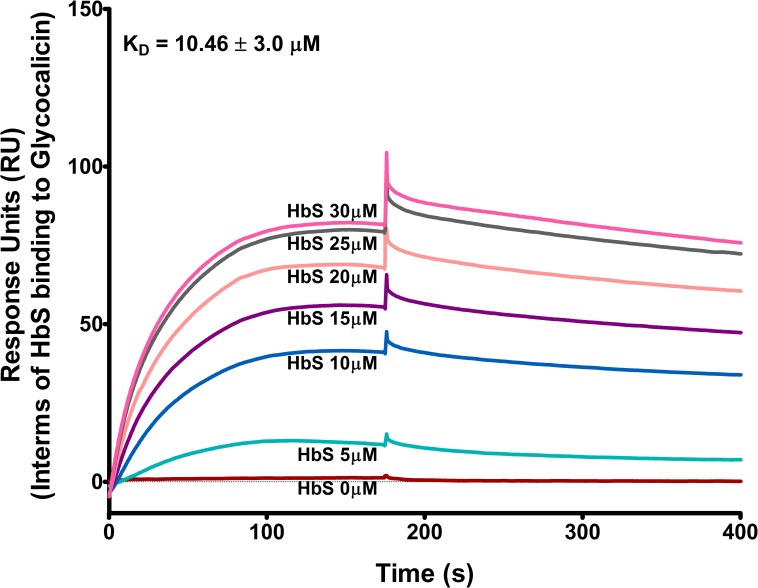
HbS binding to glycocalicin. Various concentrations of HbS was perfused at a flow rate of 30 μL/min over glycocalicin coated C1 chip and binding was measured using Surface Plasmon Resonance assay in BIAcore T200. HbS bound to glycocalicin with a binding affinity constant, K_D_ ~ 10 ± 3 μM.

Since our previous study revealed an efficient binding interaction between HbA and platelet GP1bα [13], we further investigated whether HbS interactions with platelets is mediated via HbS-GP1bα binding. Our data show that HbS bound to glycocalicin (extra membranous part of GP1bα) in a concentration-dependent manner with a binding affinity constant, K_D_ ~10.46 ± 3 μM (Fig 2), in fact with a higher affinity when compared with the HbA-glycocalicin binding, K_D_ ~44.22 ± 8.7 μM [13]. Study also investigated the HbS binding to glycocalicin in presence of haptoglobin (Hp), which makes complex with Hb; and also in presence of hemopexin Hpx), heme-binding protein, showing no effects on HbS binding to glycocalicin (S2B and S2C Fig). Further, study also measured a concentration-dependent binding of metHb to glycocalicin (S3 Fig), suggesting an interaction independent of oxidation state of Hb. Further, our data showed that metHb activated platelets and increased the expression of P-selectin and binding of PAC1 on platelet surface in a concentration-dependent manner (S4 Fig).

### HbS activates platelets via Lyn, PI3K, Akt and ERK pathway, and the activation is abrogated by the peptide AA1-50

**Fig 3:**
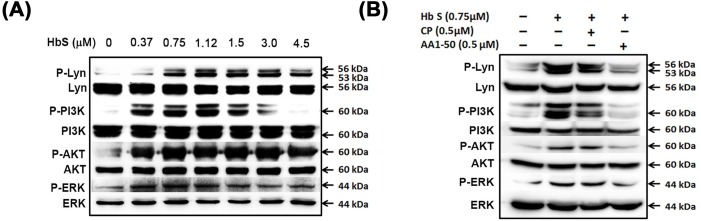
**(A) HbS activates platelets signaling proteins**. Washed platelets were incubated with various concentrations of HbS. The platelet pellet lysate was immunoblotted for phospho/non phospho-Lyn, PI3K, Akt and ERK. Data presented as the mean ± SEM fold change of the phospho/non-phospho bands from three different experiments; densitometry data are mentioned in S5 Fig The HbS (0.37–4.5 μM) increased phosphorylation of the above signaling proteins in concentration-dependent manner (for Lyn and PI3K, ***p<0.0001; Akt, *p<0.01 and **p<0.004; ERK, **p<0.001, ***p<0.0001). **(B)** The AA1-50 peptide (0.5 μM) inhibited significantly the HbS-mediated phosphorylation of the above signaling proteins (for Lyn, ***p<0.0009; PI3K, **p<0.008; Akt, *p<0.03; ERK, **p<0.004); densitometry data are mentioned in supplemental S6 Fig The control peptide (CP) did not show significant effects.

Further *in vitro* experiments show that the phosphorylation of signaling proteins such as Lyn, PI3K, Akt and ERK was increased in a concentration-dependent manner in platelets in presence of HbS (ranging between 0.37 to 4.5μM). The phosphorylation of the platelet Lyn was increased maximally at 1.5μM HbS with a fold-change 19.56±2.73 (ratio of p-Lyn/Lyn). Similarly the phosphorylation of PI3K was maximum with a fold-change of 53.44±2.47 at 1.12 μM HbS; Akt with maximum fold-change of 2.45±0.51 at 1.5 μM HbS; and ERK with a maximum fold-change of 2.51±0.13 at 1.12μM HbS (Fig 3A, S5 Fig). Furthermore, the above phosphorylation was abrogated by a synthetic peptide AA1-50, designed from N-terminal amino acids M1-T50 sequence of GP1bα. In a recent work, we have described that the peptide AA1-50 is a potent inhibitor for HbA binding to GP1bα [13]. The AA1-50 inhibited the HbS-mediated phosphorylation of Lyn, PI3K, Akt and ERK with the fold-change of 1.06±0.03, 0.711±0.13, 1.32±0.13 and 2.17±0.07 respectively (Fig 3B, S6 Fig).

### HbS-mediated platelet activation promotes thrombus formation on immobilized VWF

**Fig 4:**
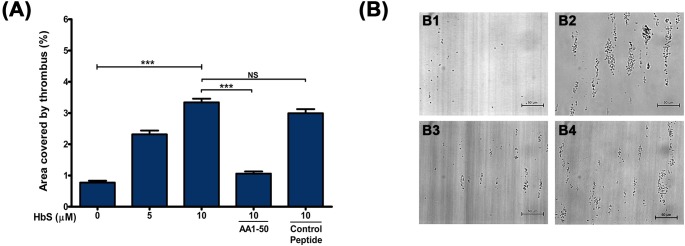
Platelet thrombus formation on immobilized VWF in the presence of HbS. **(A)** Whole blood from healthy donor was perfused over immobilized VWF (100 μg/mL) under a shear stress of 25 dyne/cm^2^ in the presence of HbS. Data are the mean ± SEM of the area covered by thrombus on VWF surface as calculated from ten fields of view in three independent experiments. The HbS increased the thrombus area in a concentration-dependent manner, ***P<0.0001. Further, the peptide AA1-50 abrogated the HbS-induced thrombus formation, ***P<0.0001. NS = non significant. **(B)** The 40X images show the platelet thrombus was increased in presence of 10 μM HbS at shear stress 25 dyne/cm^2^ **(B2)** compared to no-HbS **(B1)**. Further the thrombus was decreased in presence of 5 μM AA1-50 **(B3)** but not in presence of control peptide **(B4)**.

Our study further investigated whether the HbS-mediated activation of platelets promote thrombus formation on immobilized subendothelium matrix under flow shear stresses. The thrombus formation assay was performed *in vitro* by perfusing whole blood (from normal individuals) over immobilized VWF in the presence of HbS under shear stress, simulating arterial flow. The thrombus formation (estimated as percent area covered by thrombus) was increased in a concentration-dependent manner from 0.77±0.059 (without HbS) to 3.34±0.11 (with 10μM HbS) under shear stress of 25 dyne/cm^2^ (Fig 4A). The peptide AA1-50, which blocks the HbS/HbA binding to GP1bα, significantly reduced the thrombus formation to the value of 1.06±0.07 (Fig 4A and 4B).

### Plasma Hb concentrations correlate with platelet activation in SCD patients

**Fig 5:**
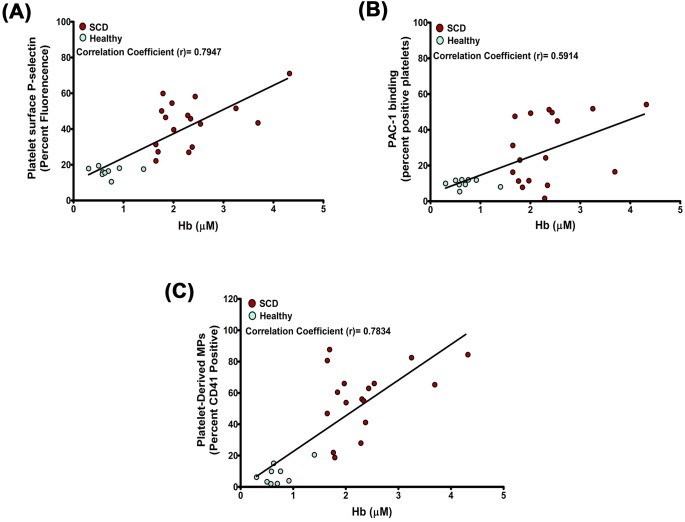
Correlation between plasma Hb and platelet activation in patients with SCD. Extracellular Hb concentration was measured from plasma of SCD patients (n = 17) and healthy individuals (n = 9) using a sandwich-ELISA. The platelet activation markers such as, P-selectin expression on platelets or PAC1 binding to platelets or MP generation by platelets was measured by flow cytometry. Plasma HbS shows positive correlations with **(A)** platelet P-selectin, **(B)** PAC1 binding and **(C)** platelet-derived MPs in plasma. Each dot represents individual data.

To assess the *in vivo* correlations between extracellular Hb and platelet activation, we estimated the plasma levels of extracellular Hb and platelet activation markers in SCD patients and normal individuals. The plasma Hb showed the positive correlation with platelet surface P-selectin (r = 0.7947, Fig 5A) as well as PAC1 binding to GPIIbIIIa (r = 0.5914, Fig 5B). Plasma Hb also correlated positively with plasma levels of platelet-derived microparticles (r = 0.7834, Fig 5C), suggesting a unique association between intravascular hemolysis and platelet activation in SCD.

## Discussion

Our study reveals that the sickle Hb (HbS, occurs due to the substitution of amino acid Glu>Val at 6^th^ position of β-globin chain of Hb) activates platelets through a similar mechanism as that of normal Hb (HbA, [13]). The HbS bound to GP1bα (Fig 2) and activated the intracellular signaling proteins of Lyn-ERK pathway in platelets (Fig 3A). The HbS concentrations (1–10 μM) commonly seen in sickle patients [8, 18] potentiated the platelet activation (Figs 1 and 3) and also promoted the platelet thrombus formation on subendothelium matrix (Fig 4) in a concentration-dependent manner *in vitro*. The HbS-GP1bα interaction initiated a series of changes in platelets including granule secretion (Fig 1A), expression of surface integrin GPIIbIIIa (Fig 1B) and shape change (Fig 1D). The HbS-GP1bα interaction also triggered the inside out signaling processes in platelets via Lyn-ERK pathway (Fig 3A) as described for HbA-GP1bα [13] or VWF-GP1bα interaction [19, 20]. This interaction has also been seen to increase the platelet thrombus formation on VWF surface under flow condition (Fig 4). The HbS-induced activation of platelets probably played a crucial role in stabilizing the thrombus as observed for normal Hb (HbA, [14].

Furthermore the inhibition of HbS-mediated platelet activation or thrombus formation by the synthetic peptide AA1-50 (Figs 3B and 4) confirms the role of HbS-GP1bα interaction in platelet functions and also suggests that the interaction site for HbS probably exits between amino acid Met 1 and Tyr 50 of the N-terminal domain of GP1bα. We have reported earlier the similar site for HbA binding to GP1bα as the interaction of the two proteins was abrogated by AA1-50 [13].

The concentration of free Hb in plasma appears to be the crucial factor for platelet activation contributing to the pathogenesis of thrombosis and procoagulant states in hemolytic disorders. The effects of extracellular Hb on platelet function are further supported by data from patients with SCD; a hemolytic disorder characterized by the high propensity to vascular occlusion and hypercoagulable states [2, 9]. Our data showed a direct correlation of plasma extracellular Hb with platelet activation markers such as surface P-selectin expression and PAC1 binding, and also with platelet-derived microparticles in plasma (Fig 5) of sickle patients, similar to the phenomenon described recently by us in PNH patients [13]. Our study therefore suggests that HbS can regulate platelet functions and potentiate the thrombotic as well as pro-coagulation complications in SCD.

## Supporting Information

S1 Fig Adenosin diphosphate (ADP) was estimated in HbS (0, 0.5, 3, 4.5, 7.5 and 9 μM) and hemolysate of sickle RBCs using ADP colorimetric assay kit. **(A)** Standard plot was plotted against the different concentrations of ADP standard solution against the absorbance at 570nm. **(B)** The levels of ADP in HbS solution and hemolysate were calculated with the help of standard plot as instructed in the kit and data were represented as Mean ± SD.
(TIF)

S2 Fig HbS binding to glycocalicin in presence of haptoglobin (Hp) and hemopexin (Hpx). Different concentrations of HbS (0, 0.25, 0.5, 1, 3 and 5μM) were incubated in glycocalicin (10 μg/mL) coated ELISA plate. **(A)** The HbS binding was detected using anti-Hbα antibody and anti-goat IgG HRP conjugated antibody at O.D.~450nm. HbS bound to glycocalicin in dose dependent manner, ***P<0.0001. **(B)** Further, HbS (5μM) binding to glycocalicin was measured in presence of various concentrations of Hp (0 μM, 1 μM, 2.5 μM, 5μM) and **(C)** Hpx (0 μM, 1 μM, 2.5 μM, 5μM). Data show no effects of either Hp or Hpx on the HbS-glycocalicin binding.
(TIF)

S3 Fig MetHb binding to glycocalicin. Various concentrations of MetHb (0 μM, 0.25 μM, 0.5 μM, 1 μM, 3 μM, 5μM) were incubated in glycocalicin (10 μg/mL) coated ELISA plate. MetHb binding was detected using HRP tagged anti-Hb antibody at O.D.~450nm. MetHb bound to glycocalicin in a concentration-dependent manner, **P<0.001, ***P<0.0001.
(TIF)

S4 Fig Platelet activation in presence of MetHb. **(A)** Washed platelets were incubated with various concentrations of MetHb (0, 0.5, 1.0, 1.5, 2.0, 3.0, 4.5, 9.0, 12.5, and 15μM) and labeled with anti-P selectin FITC antibody for flow cytometry measurement. HbS increased the P-selectin expression in a concentration-dependent manner, ***P<0.0001. **(B)** The PAC1 binding to platelets GPIIbIIIa was measured using flow cytometry. The HbS increased the PAC1 binding in a concentration dependent manner, ***P<0.0001, *P<0.01.
(TIF)

S5 Fig Densitometry ratio of phospho /non-phospho **(A)** Lyn, ***P<0.0001 (compared to HbS 0μM); **(B)** PI3K, ***P<0.0001(compared to HbS 0μM); **(C)** AKT, *P<0.01, **P<0.004 (compared to HbS 0μM) and **(D)** ERK, **P<0.001, ***P<0.0001 (compared to HbS 0μM), as mentioned in Fig 3A. Data are the mean ± SEM fold change from three experiments.
(TIF)

S6 Fig Densitometry ratio of phospho /non-phospho **(A)** Lyn, ***P<0.0009, *P<0.03, (compared to HbS 0.75 μM.); **(B)** PI3K, **P<0.008, NS = non-significant (compared to HbS 0.75 μM.); **(C)** AKT, *P<0.03, NS = non-significant, (compared to HbS 0.75 μM.); and **(D)** ERK, **P<0.004, NS = non-significant, (compared to HbS 0.75 μM.), as mentioned in Fig 3B. Data are the mean ± SEM fold change from three experiments.
(TIF)

S1 File (DOCX)
