# Supplementary figures and images for: HbS Binding to GP1bα Activates Platelets in Sickle Cell Disease
Source: PLoS One. 2016 Dec 9;11(12):e0167899. doi: 10.1371/journal.pone.0167899 (PMC5148012; doi:10.1371/journal.pone.0167899)

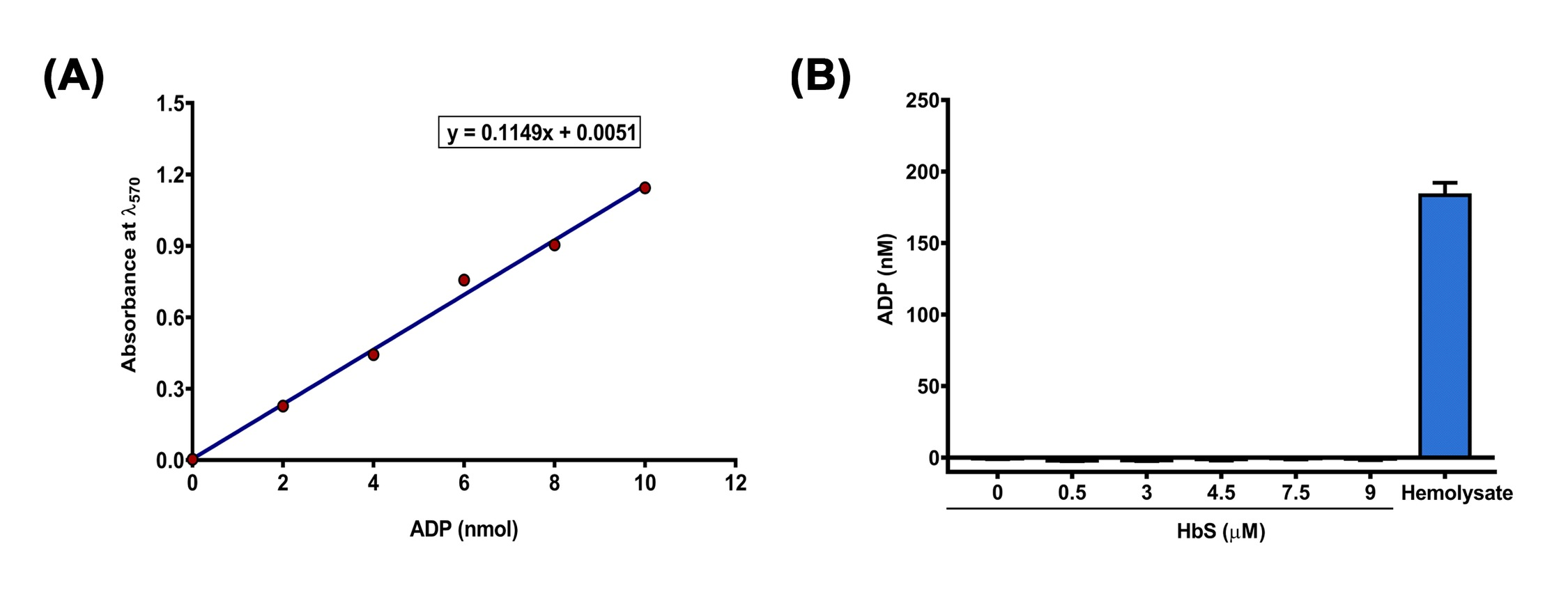

Supplement: S1 Fig — (A) Standard plot was plotted against the different concentrations of ADP standard solution against the absorbance at 570nm. (B) The levels of ADP in HbS solution and hemolysate were calculated with the help of standard plot as instructed in the kit and data were represented as Mean ± SD. (TIF) [file pone.0167899.s001.tif]

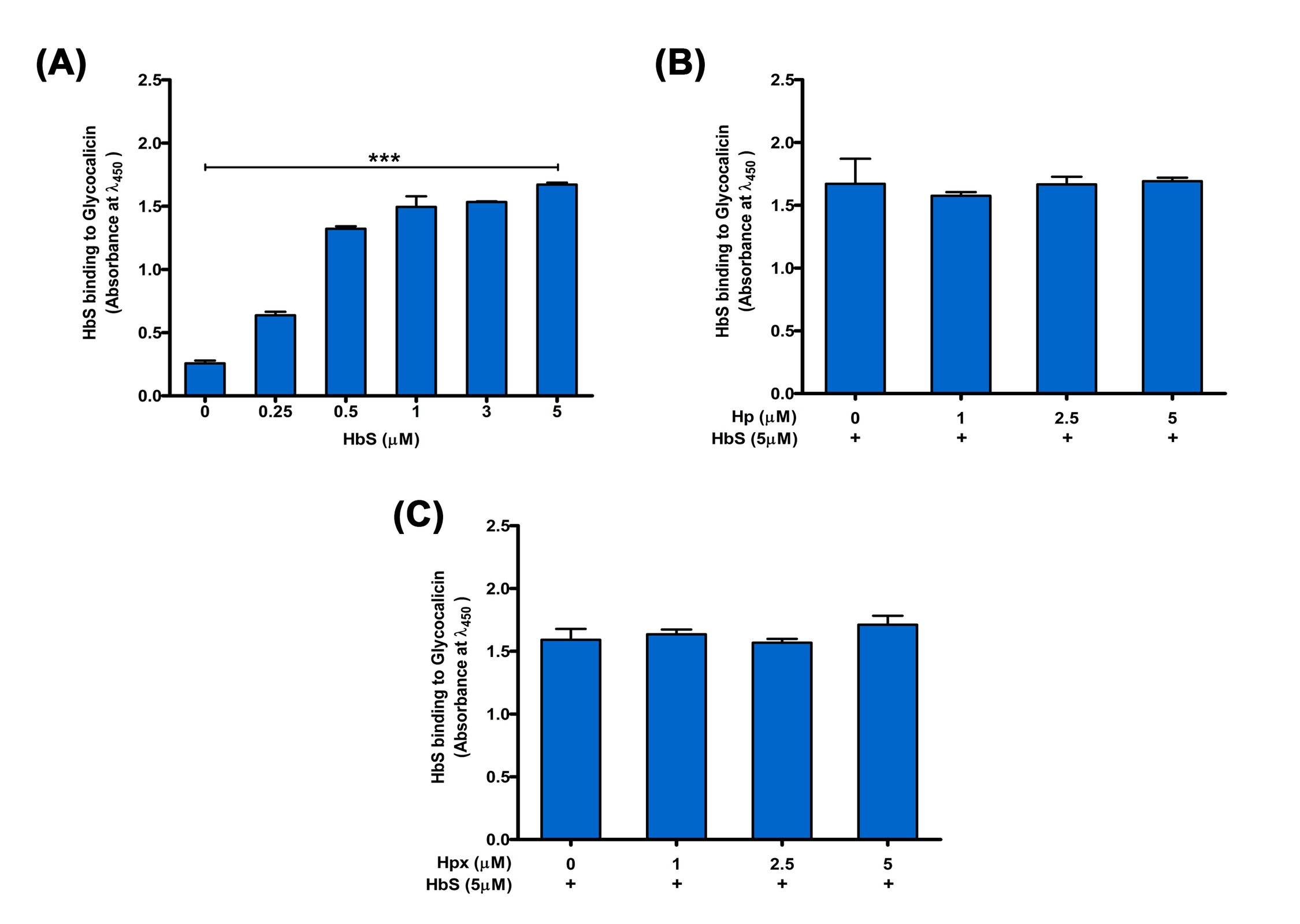

Supplement: S2 Fig — Different concentrations of HbS (0, 0.25, 0.5, 1, 3 and 5μM) were incubated in glycocalicin (10 μg/mL) coated ELISA plate. (A) The HbS binding was detected using anti-Hbα antibody and anti-goat IgG HRP conjugated antibody at O.D.~450nm. HbS bound to glycocalicin in dose dependent manner, ***P<0.0001. (B) Further, HbS (5μM) binding to glycocalicin was measured in presence of various concentrations of Hp (0 μM, 1 μM, 2.5 μM, 5μM) and (C) Hpx (0 μM, 1 μM, 2.5 μM, 5μM). Data show no effects of either Hp or Hpx on the HbS-glycocalicin binding. (TIF) [file pone.0167899.s002.tif]

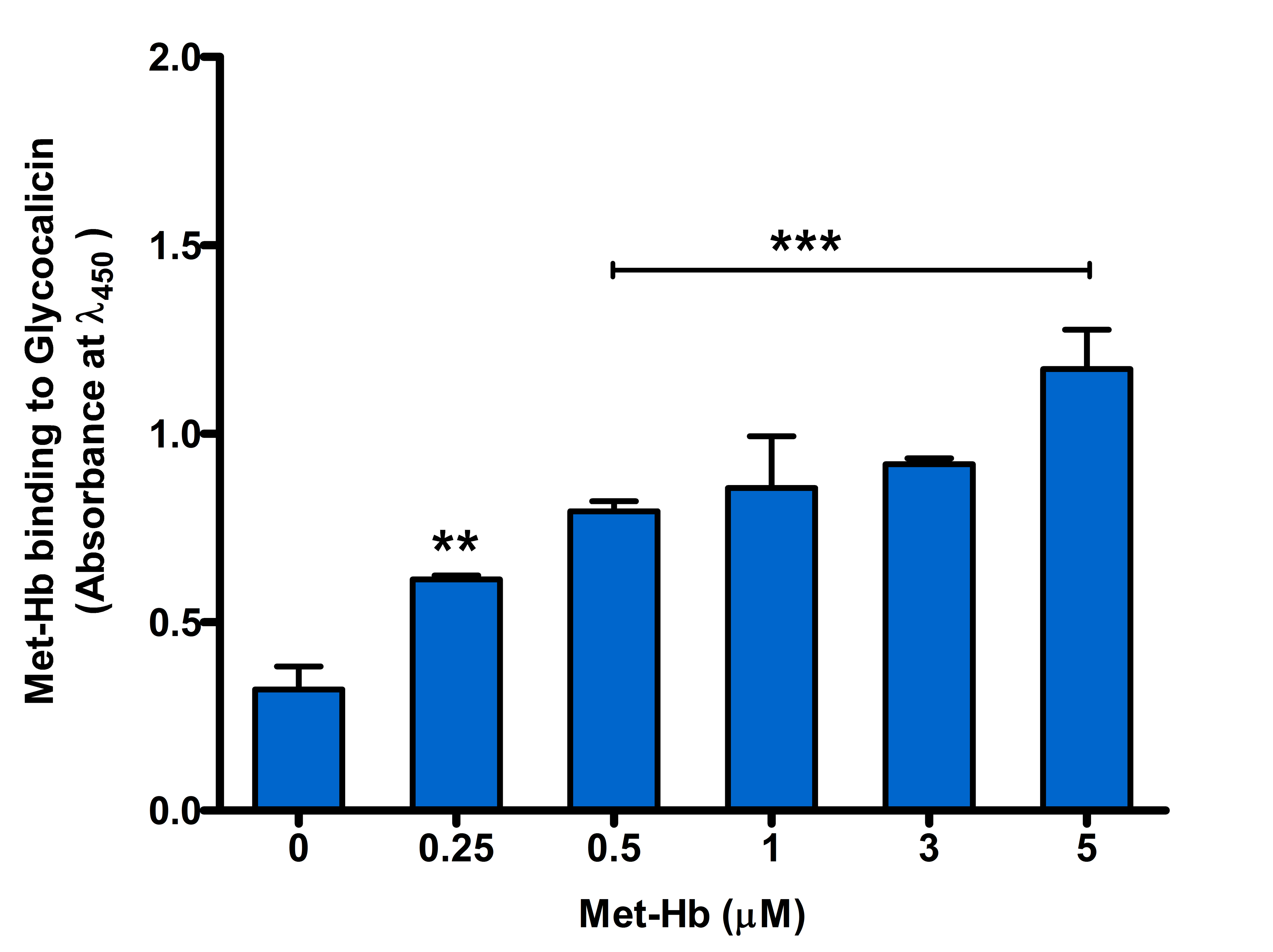

Supplement: S3 Fig — Various concentrations of MetHb (0 μM, 0.25 μM, 0.5 μM, 1 μM, 3 μM, 5μM) were incubated in glycocalicin (10 μg/mL) coated ELISA plate. MetHb binding was detected using HRP tagged anti-Hb antibody at O.D.~450nm. MetHb bound to glycocalicin in a concentration-dependent manner, **P<0.001, ***P<0.0001. (TIF) [file pone.0167899.s003.tif]

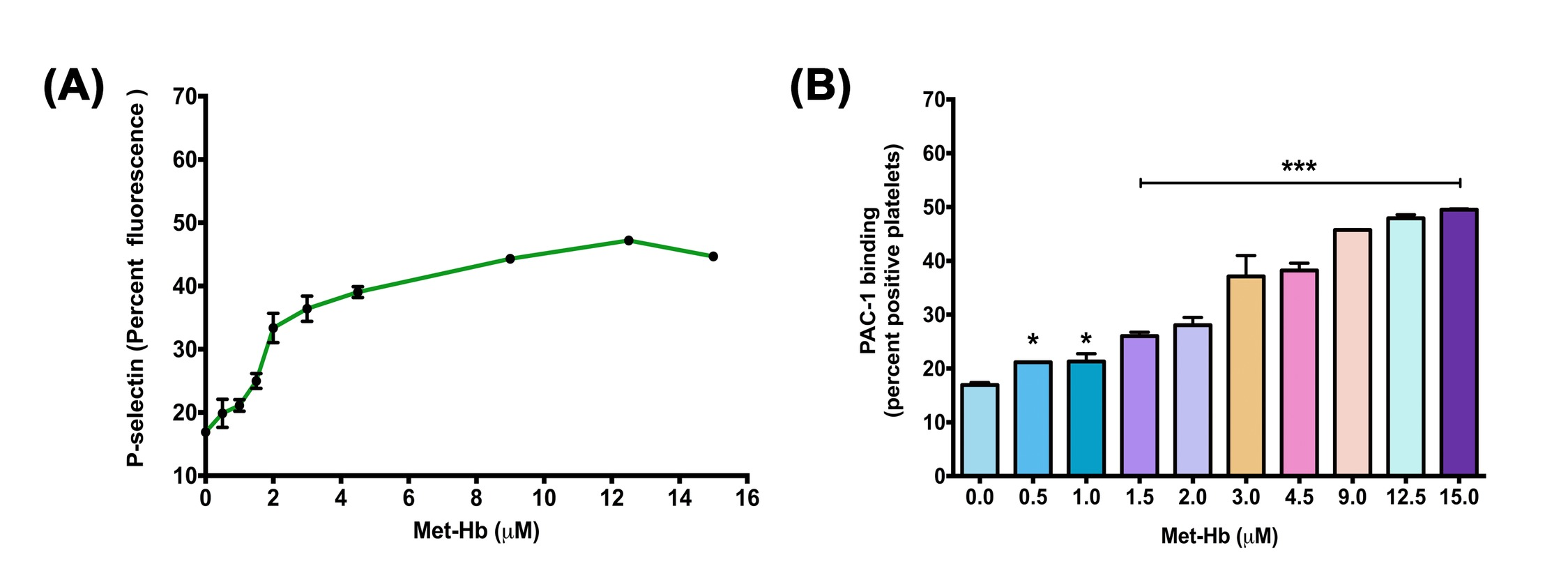

Supplement: S4 Fig — (A) Washed platelets were incubated with various concentrations of MetHb (0, 0.5, 1.0, 1.5, 2.0, 3.0, 4.5, 9.0, 12.5, and 15μM) and labeled with anti-P selectin FITC antibody for flow cytometry measurement. HbS increased the P-selectin expression in a concentration-dependent manner, ***P<0.0001. (B) The PAC1 binding to platelets GPIIbIIIa was measured using flow cytometry. The HbS increased the PAC1 binding in a concentration dependent manner, ***P<0.0001, *P<0.01. (TIF) [file pone.0167899.s004.tif]

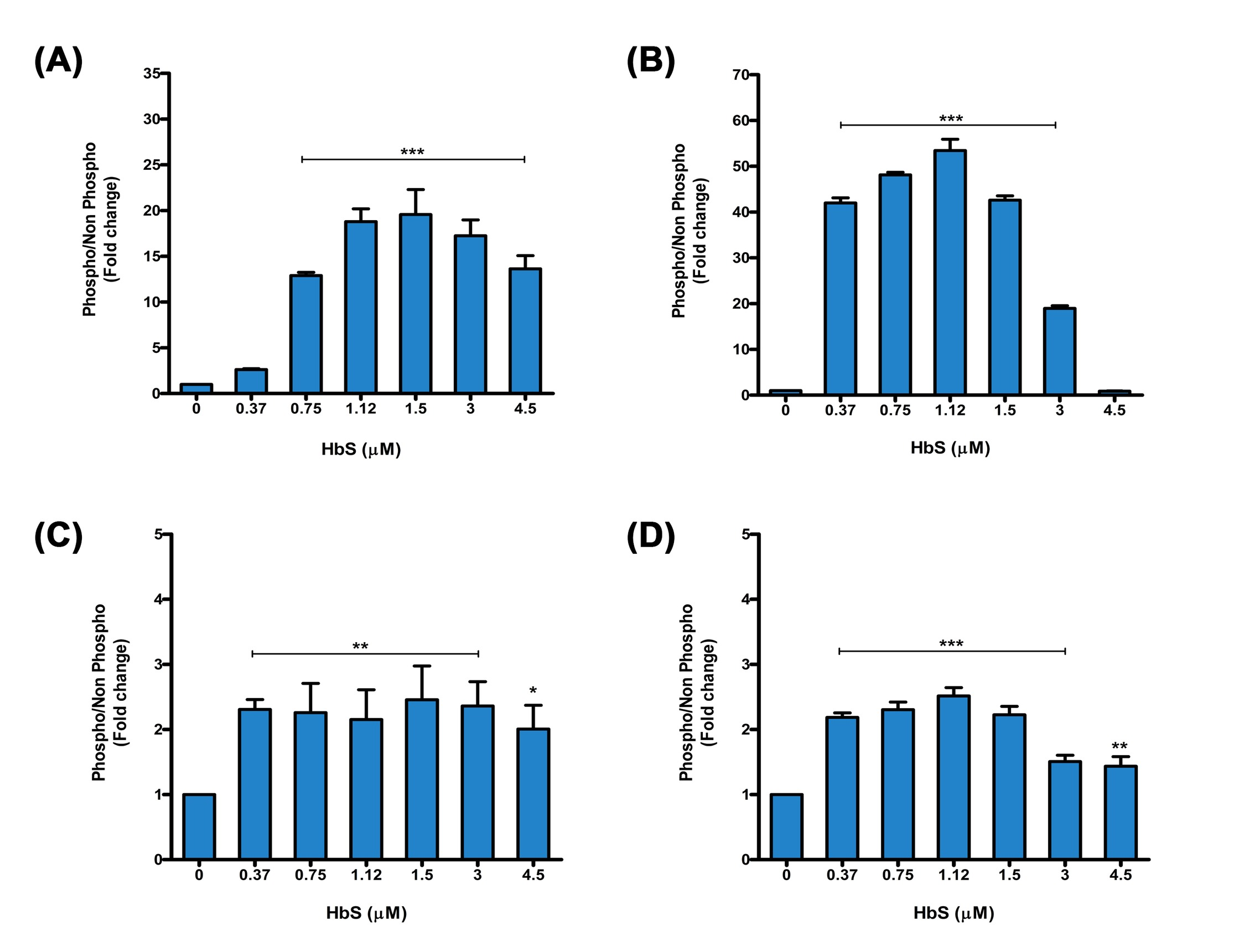

Supplement: S5 Fig — Densitometry ratio of phospho /non-phospho (A) Lyn, ***P<0.0001 (compared to HbS 0μM); (B) PI3K, ***P<0.0001(compared to HbS 0μM); (C) AKT, *P<0.01, **P<0.004 (compared to HbS 0μM) and (D) ERK, **P<0.001, ***P<0.0001 (compared to HbS 0μM), as mentioned in Fig 3A. Data are the mean ± SEM fold change from three experiments. (TIF) [file pone.0167899.s005.tif]

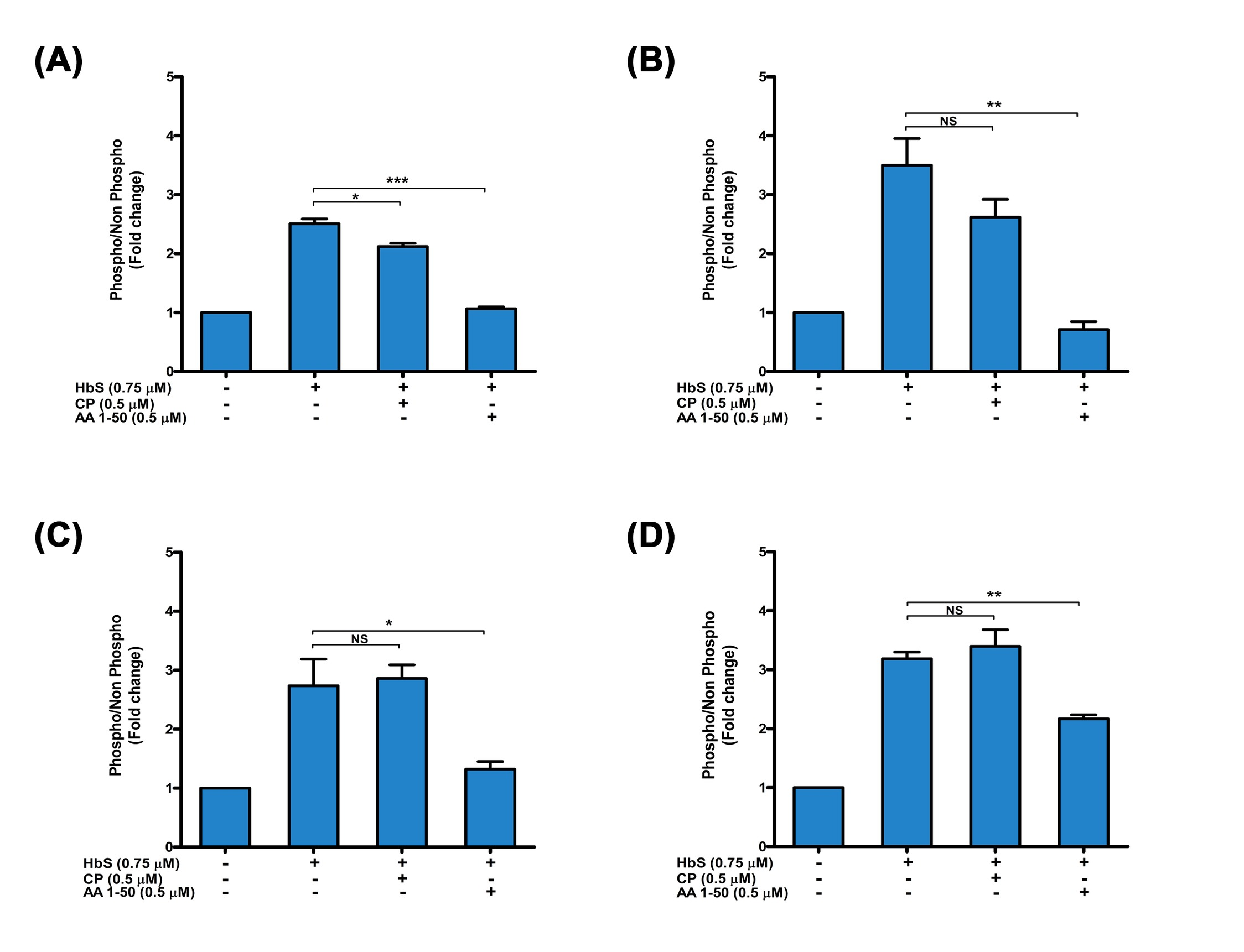

Supplement: S6 Fig — Densitometry ratio of phospho /non-phospho (A) Lyn, ***P<0.0009, *P<0.03, (compared to HbS 0.75 μM.); (B) PI3K, **P<0.008, NS = non-significant (compared to HbS 0.75 μM.); (C) AKT, *P<0.03, NS = non-significant, (compared to HbS 0.75 μM.); and (D) ERK, **P<0.004, NS = non-significant, (compared to HbS 0.75 μM.), as mentioned in Fig 3B. Data are the mean ± SEM fold change from three experiments. (TIF) [file pone.0167899.s006.tif]
