# Supplementary material for: HbS Binding to GP1bα Activates Platelets in Sickle Cell Disease
Source: PLoS One. 2016 Dec 9;11(12):e0167899. doi: 10.1371/journal.pone.0167899 (PMC5148012; doi:10.1371/journal.pone.0167899)
Supplement: S1 File — (DOCX) [file pone.0167899.s007.docx]

**Supplemental methods**

**Methemoglobin (MetHb)**: The human recombinant HbA was generous gift from Dr. Suman Kundu, Delhi University, India; was generated following the methods as described [[1](#_ENREF_1)]. The MetHb was prepared by treating the recombinant Hb with potassium ferricyanide (Sigma Aldrich, St. Louis, USA) in 5:1 ratio for 10 minutes then subjected to Sephadex G-25 and dialyzed against PBS for the removal of ferricyanide [[2](#_ENREF_2)].

**ELISA:** The binding of MetHb to glycocalicin was detected using ELISA. ELISA plate was coated with glycocalicin (10µg/mL) and blocked with 2% BSA. Various concentrations of MetHb (0.25µM, 0.5 µM, 1 µM, 3 µM and 5 µM) were incubated at 37^o^C for an hour separately. Binding of MetHb to Glycocalicin was detected using HRP tagged anti-Hb antibody (Abcam, Cambridge, MA, USA). The OD was measured at 450 nm.

**Flow cytometry:** Flow cytometry was performed to detect the activation markers on platelet surface including P-selectin expression and PAC1 binding. Washed platelets treated with various concentrations of MetHb and were labelled with anti-P-selection FITC or PAC1 FITC antibody. The surface expression of P-selectin and PAC1 binding on platelets was measured by flow cytometry (Becton Dickinson, San Jose, CA, USA).

**ADP estimation:** ADP was measured in the solution of HbS (Sigma Aldrich, St. Louis, USA) and lysate of sickle RBCs using ADP colorimetric assay kit (BioVision, CA, USA) by following the manufacturer’s protocol. Different concentrations of HbS (0µM, 0.5µM, 3µM, 4.5µM, 7.5µM, 9µM) or lysate from sickle RBCs were incubated with reaction mix (mixture of ADP probe, ADP converter, ADP developer and assay buffer) for 30 minutes at room temperature and the absorbance was measured at 570 nm. Similarly a standard plot from different concentrations of ADP standard (supplied with kit) was made and intrapolated by employing linear regression curve fit. The amount of ADP present in the solution of HbS and lysate of sickle RBCs were calculated from the standard plot.

**Supplemental References**

1. Graves PE, Henderson DP, Horstman MJ, Solomon BJ, Olson JS (2008) Enhancing stability and expression of recombinant human hemoglobin in E. coli: Progress in the development of a recombinant HBOC source. Biochim Biophys Acta 1784: 1471-1479.

2. Helms CC, Marvel M, Zhao W, Stahle M, Vest R, et al. (2013) Mechanisms of hemolysis-associated platelet activation. J Thromb Haemost 11: 2148-2154.
